# Tight Junction Protein Signaling and Cancer Biology

**DOI:** 10.3390/cells12020243

**Published:** 2023-01-06

**Authors:** Zeina Nehme, Natascha Roehlen, Punita Dhawan, Thomas F. Baumert

**Affiliations:** 1 Université de Strasbourg, Inserm, Institut de Recherche sur les Maladies Virales et Hépatiques UMR_S1110, 67000 Strasbourg, France; 2 Department of Medicine II (Gastroenterology, Hepatology, Endocrinology and Infectious Diseases), Freiburg University Medical Center, Faculty of Medicine, University of Freiburg, 79098 Freiburg, Germany; 3 Department of Biochemistry and Molecular Biology, University of Nebraska Medical Center, Omaha, 68198 NE, USA; 4 Buffet Cancer Center, University of Nebraska Medical Center, Omaha, 68105 NE, USA; 5 VA Nebraska-Western Iowa Health Care System, Omaha, 68105-1850 NE, USA; 6 Institut Hospitalo-Universitaire (IHU), Pôle Hépato-Digestif, Hôpitaux Universitaires de Strasbourg, 67000 Strasbourg, France; 7 Institut Universitaire de France, 75006 Paris, France

**Keywords:** tight junctions, carcinogenesis, signaling pathways, therapeutic targets

## Abstract

Tight junctions (TJs) are intercellular protein complexes that preserve tissue homeostasis and integrity through the control of paracellular permeability and cell polarity. Recent findings have revealed the functional role of TJ proteins outside TJs and beyond their classical cellular functions as selective gatekeepers. This is illustrated by the dysregulation in TJ protein expression levels in response to external and intracellular stimuli, notably during tumorigenesis. A large body of knowledge has uncovered the well-established functional role of TJ proteins in cancer pathogenesis. Mechanistically, TJ proteins act as bidirectional signaling hubs that connect the extracellular compartment to the intracellular compartment. By modulating key signaling pathways, TJ proteins are crucial players in the regulation of cell proliferation, migration, and differentiation, all of which being essential cancer hallmarks crucial for tumor growth and metastasis. TJ proteins also promote the acquisition of stem cell phenotypes in cancer cells. These findings highlight their contribution to carcinogenesis and therapeutic resistance. Moreover, recent preclinical and clinical studies have used TJ proteins as therapeutic targets or prognostic markers. This review summarizes the functional role of TJ proteins in cancer biology and their impact for novel strategies to prevent and treat cancer.

## 1. Tight Junction Proteins Exhibit a Large Repertoire of Functional Roles in Cell Biology

Described as intercellular adhesion complexes in epithelia and endothelia, tight junction (TJ) proteins constitute a multi-functional machinery that regulates paracellular permeability and cell polarity [1]. TJ proteins are classified based on multiple genetic and molecular functions with three transmembrane proteins that are common to all tight junctions [2]. These three proteins comprise claudins (CLDNs), the tight junction-associated MARVEL domain proteins, including occludin, tricellulin (also known as the MARVEL domain-containing protein 2 (MARVELD2)) and MARVELD3, in addition to the junction adhesion molecule (JAM) [3]. Other commonly expressed transmembrane TJ proteins include the Crumbs proteins (CRBs), the angulin proteins, the coxsackievirus–adenovirus receptor (CAR), and BVES (blood-vessel epicardial substance, also known as POPDC1 for Popeye domain-containing protein-1) [4]. Furthermore, the region of the cytoplasm that underlies the tight junctions harbors several multi-molecular protein complexes [5]. The latter include proteins associated with Lin Seven 1 (Pals1), multi-PDZ domain protein 1 (MUPP1), Zona occluden-1, -2 and -3 (ZO-1, ZO-2; ZO-3), and the non-PDZ-expressing proteins, including cingulin, symplekin, ZO-1-associated nucleic acid-binding protein (ZONAB), GEF-H1, aPKC, PP2A, Rab3b, Rab13, PTEN, and 7 H6 [5].

Two types of barrier functions are described to be mediated by TJs, namely the gate and the fence functions [1]. Indeed, the regulation of paracellular permeability is one of the major physiological functions of TJs, as this controls the transport of molecules across tissues, and between different body compartments [6]. In this context, selective permeability is tightly controlled by size and charge. The size-selective pathway allows the diffusion of macromolecules up to a size limit of ∼30–60 Å, in contrast to the charge-selective small-pore pathway with pore diameters of ∼4–8 Å [7]. On the other hand, TJ proteins are involved in the fence function, also known as the intramembrane diffusion barrier, which refers to the segregation of the apical and basolateral plasma membranes [8].

**Figure 1:**
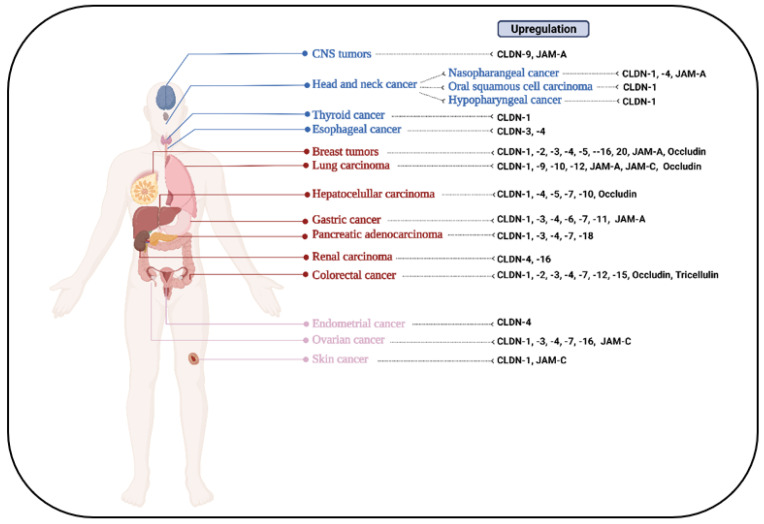
Examples of upregulation of tight junction protein expression in various tumor types. CLDN: claudin; JAM: junction adhesion molecule.

Beyond their classical functions, TJ proteins also modulate polarity, differentiation, growth and proliferation, as well as cell migration and motility, as these molecules act as arbitrators and transducers of cell-to-cell adhesion and signaling cascades [9]. Consequently, TJ protein alteration during pathological processes has been described to interfere with the proper functioning of the cellular machinery and homeostasis maintenance [10]. In cancer, changes in the expression and/or localization of these molecules are frequently reported (Figure 1) [11]. However, many of these studies are observational, and well-founded mechanistic studies that link these multi-component complexes to disease pathogenesis are lacking [12]. Dissecting the role of TJ complexes in various aspects of oncogenic transformation could catalyze our conception of TJ proteins as bidirectional signaling hubs and advance our perception of their multifunctional nature as an inherent player of the evolutionarily conserved signaling cascades. In this context, this review aims to tackle the interrelation between TJ components and signal transduction in cancer pathophysiology from a molecular, rather than a cellular biology perspective. Considering the frequent dysregulation in malignant diseases, we further discuss TJ components as targets of anti-cancer treatments.

## 2. From Intercellular Adhesion and Polarity Regulators to Signaling Modulators in Cancer Biology

The complexity of TJ functionality is highlighted by their dual and differential expression patterns in different cancer types, where loss of expression, or alternatively overexpression, are described to promote tumorigenesis [13]. This is further confirmed in several in vivo animal models, where the aberrant expression of TJ proteins promotes abnormal cellular proliferation, neoplasia, or metastasis [11]. At a glance, 5297 abstracts were identified by Vermette and coworkers as potential articles that emphasize the relationship between biomarkers of endothelial or epithelial TJ structures or functions and critical illness [14]. In this setting, recent data have repositioned the perception of TJ proteins from a simple static constituent of junctions to multi-component and multi-functional signaling complexes involved in the regulation of a multitude of cellular events [15]. For example, the 44 amino acids (aa) at the COOH-terminal end of occludin were shown to play a critical role in receiving and transmitting intracellular apoptosis-inducing signals [16]. Of note, beside modulating established signaling cascades, some TJ-associated proteins can translocate to the nucleus where they regulate gene expression [17].

Among the complex events that mediate oncogenesis, alterations in signal transduction pathways are the most commonly described [18]. Recently, through an integrated analysis of The Cancer Genome Atlas (TCGA), Sanchez-Vega and coworkers emphasized individual and co-occurring actionable alterations in the 10 main oncogenic pathways as potential drivers and functional contributors of oncogenic transformation [19]. This repertoire of analyzed pathways includes the following: (1) cell cycle alterations, (2) Hippo signaling, (3) Myc signaling, (4) Notch signaling, (5) phosphatidylinositol-3 kinase (PI3K) signaling, (6) oxidative stress response, (7) receptor-tyrosine kinase (RTK)/RAS/mitogen-activated protein kinase (MAPK) signaling, (8) transforming growth factor-beta (TGF-β) signaling, (9) Wnt/β-catenin signaling, and (10) p53 [19]. Interestingly, interactions between tight junction components and these pathways have been described. The following section discusses illustrative examples of various TJ proteins crosstalks with the aforementioned pathways, with a detailed mapping of these signaling cascades being developed in the subsequent section. Regarding cell cycle alteration, ZONAB, which is known to bind to the SH3 domain of ZO-1, was shown to regulate cellular proliferation and G1/S phase progression through interacting with the cyclin-dependent kinase 4 (CDK4) [20]. Intriguingly, ZO-2-mediated transcriptional repression of cyclin D1 and the subsequent cell proliferation alteration was suggested to be mediated by the interaction with c-Myc [21]. The latter was also studied in the context of CLDN perturbation, where CLDN7 knockdown increased c-Myc expression at the transcriptional and proteomic levels [22]. CLDN1 overexpression, on the other hand, increased c-Myc levels in colon cancer cells [23]. Regarding oncogenic pathways, CLDN18 overexpression was shown to decrease Yes-associated protein (YAP) nuclear localization and transcriptional activity, whereas its loss decreased YAP interaction with Hippo kinases p-LATS1/2 [24]. In addition, CLDN18 and YAP were shown to interact and colocalize at cell–cell contact, with CLDN18 being identified as a YAP regulator [24]. Beside the Hippo/YAP pathway, other oncogenic signaling cascades were described to be modulated by TJ proteins. For instance, by inducing matrix metalloproteinase-9 (MMP-9) and extracellular signal-regulated kinase (ERK) signaling, CLDN1 regulates Notch signaling and cellular differentiation [25], as well as β-catenin pathway activation, mediating colitis-associated cancer [26]. Beside CLDNs, occludin was also described to interact with other signaling pathways, for instance the PI3K cascade. Indeed, the latter interacts with occludin through its C-terminal tail, where p85, the regulatory subunit of PI3K, co-immunoprecipitates with occludin [27]. Specifically, a 27 aa peptide of the coiled coil domain of occludin was identified to act as a site for specific interactions with several potential regulatory proteins [28]. Of note, oxidative stress increases the association of PI3K with occludin [27]. Beside PI3K, the activity of Ras effectors, including Raf kinases, is described to contribute to the sequential activation of the ERK/MAPK pathway. In this context, occludin may act as a pivotal signaling molecule, where its overexpression inhibits anchorage-independent growth and Raf-1-mediated transformation of salivary gland epithelial cells [29]. In addition, occludin extracellular loop 2 regulates the localization of TGFÎ^2^, the TGF-β type I receptor responsible for inducing the dissolution of tight junctions and acquisition of a mesenchymal phenotype [30]. Taken together, an intimate link appears to bridge TJ proteins to the well-established signaling pathways in carcinogenesis. Indeed, various TJ components crosstalk with a myriad of signaling pathways by directly modulating these cascades or through intermediate proteins, such as kinases and phosphatases. In addition, TJ proteins conserve the ability to rapidly modulate their functional properties and permeability in response to oncogenic stimuli. These diverse regulatory mechanisms not only allow the fine tuning and transmission of external signals to the cell interior, but also position TJ protein dynamics as a key aspect in cancer initiation and progression.

## 3. Tight Junction Proteins as Central Mediators of Cancer Hallmarks

**Figure 2:**
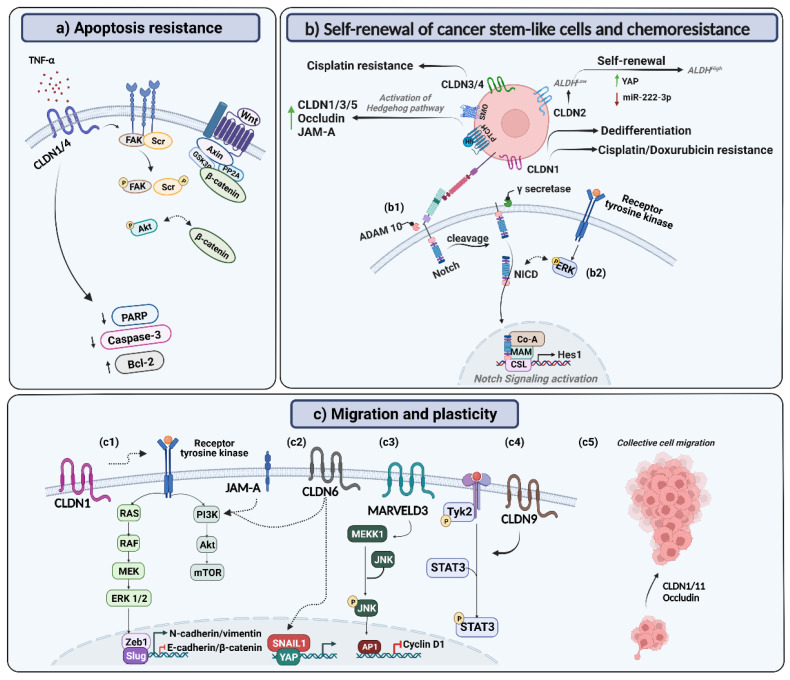
Schematic representation of examples of mechanisms mediated by members of the tight junction protein family during carcinogenesis. (**a**) Tight junction proteins are identified as potential mediators of apoptosis/anikiosis resistance. This is mainly mediated through the upregulation of caspase-3, PARP and Bcl-2, in parallel to the modulation of Src and Akt phosphorylation, coupled to β-catenin overexpression. (**b**) Tight junction proteins are also potential mediators of cancer stem-like phenotype acquisition, where they contribute to self-renewal and dedifferentiation, as well as therapeutic resistance and poor prognosis. In this setting, CLDN1 overexpression increases Notch signaling (**b1**), in parallel to the induction of MMP-9 expression and p-ERK signaling (**b2**), as well as through its non-canonical role in regulating Notch/PI3K/Wnt/β-cateninSer552 signaling. (**c**) Beside stemness, tight junction proteins are crucial regulators of cell migration and plasticity, where they activate key signaling pathways related to epithelial-to-mesenchymal transition (EMT). Indeed, CLDN1 was described to promote mesenchymal transformation by upregulating Slug and Zeb1 through the activation of the c-Abl-Ras-Raf-1-ERK1/2 signaling axis (**c1**). CLDN6 was also described to promote EMT by increasing YAP1 nuclear translocation and enhancing its interaction with Snail1 (**c2**), as well as through the activation of the PI3K/Akt pathway, along with JAM-A. MarvelD3 has been suggested to regulate migration through the JNK pathway (**c3**), and CLDN9 was shown to promote invasive cellular behavior by activating the Tyk2/STAT3 signaling pathway (**c4**). TJ proteins were also described to promote collective cell migration (**c5**). ALDH: aldehyde dehydrogenase; CLDN: claudin; JAM: junction adhesion molecule; NCID: Notch intracellular domain; PARP: poly (ADP-ribose) polymerase; TNF-α: tumor necrosis factor-alpha.

The following section thoroughly examines the functional crosstalk between a variety of TJ components and the myriad of signaling pathways that control cell dynamics, in terms of survival, migration, epithelial-to-mesenchymal transition and stemness (Figure 2). The role of TJ components in epigenetic cell regulation is also discussed.

### 3.1. Modulation of Proliferation and Apoptosis Resistance in Cancer Initiation and Progression

Standing as the most fundamental trait of cancer cells, sustained proliferative signaling is the first cancer hallmark among others to support tumor development and progression [31]. Emerging evidence points toward TJ proteins as essential regulators of cell proliferation through multiple mechanisms, including, but not limited to, microenvironment alteration, transcriptional regulation, and alteration in the cellular localization of cell cycle control proteins [32]. In this context, knockdown of the tight junction protein 1 (TJP1) gene expression significantly decreased bladder cancer cells’ growth, via dysfunction of the miR-455-TJP1 axis, where the latter suppressed TJP1 expression by directly targeting its 3′-untranslated region [33]. Another microRNA, miR-497, was also described to inhibit cell proliferation upon decreased CLDN2 expression. Mechanistically, treatment with the HDAC inhibitors trichostatin A and sodium butyrate decreases the stability of CLDN2 mRNA through the elevation of miR-497 [34]. CLDN2 expression increased cellular proliferation, anchorage-independent growth and tumor growth in vivo, potentially via the epidermal growth factor receptor (EGFR) transactivation, a key regulator of colorectal carcinogenesis [35]. Furthermore, inhibition of cell proliferation in lung adenocarcinoma is partly related to the decrease in CLDN2 expression upon treatment with the DNA methyltransferase inhibitor azacitidine (AZA) [34]. This is explained by the decrease in NF-κB phosphorylation and binding to the promoter region of CLDN2 [36]. Besides, CLDN2 retains its ability to complex with ZO-1, ZONAB, and cyclin D1, sequestering them in the nucleus, which results in enhanced cellular proliferation in lung adenocarcinoma [37]. Moreover, lipidoid-formulated CLDN3 siRNA intratumoral and intraperitoneal injections significantly reduced cell proliferation in OVCAR-3 xenografts, and tumor burden in MISIIR/TAg transgenic mice, respectively [38]. In addition, CLDN4 overexpression increased MCF-7 cells’ proliferation, with tumor size in nude mice transplanted with CLDN4-silenced MCF-7 cells being reduced [39].

Cell cycle regulators/effectors and apoptotic stimuli link cellular proliferation to apoptosis, with the dysregulation of this programmed cell death being one of the leading drivers of tumorigenesis [40]. For example, it was shown that CLDN1 knockdown couples cycle arrest to apoptosis due to the upregulation of β-catenin expression [41]. In this context, the functional role of TJ proteins has been described in apoptosis, with their disruption considered as an early event that initiates caspase activation and cell death through the interaction of occludin and CLDNs with the extrinsic apoptotic signaling pathway [42]. Indeed, CLDN1 exhibits anti-apoptotic effects under tamoxifen or TNF-α treatment in MCF-7 cells, with its knockdown increasing the expression of caspase-8 and cleaved poly (ADP-ribose) polymerase (PARP) [41,43]. An anti-apoptotic function of CLDN1 was also corroborated in nasopharyngeal carcinoma cell lines under serum deprivation or 5-fluorouracil treatment [44]. Furthermore, caspase-3 activation was increased following treatment with the apoptotic inducer staurosporine upon CLDN4 expression loss in ovarian tumor cells [45]. In line with these findings, CLDN4 overexpression reduced the rate of cell apoptosis [39]. On the other hand, resistance to anikois, an apoptosis subtype, is a critical prerequisite for carcinoma progression [46]. CLDN1 expression confers resistance to anoikis in colon cancer cells. This is mainly dependent on the activation of Src, a tyrosine kinase identified to promote anoikis resistance, with which CLDN1 was described to form a multiprotein complex, along with ZO-1 [47]. This results in the modulation of Akt phosphorylation and increased Bcl-2 expression. Furthermore, CLDN1-mediated anoikis resistance and Scr involvement were also described in gastric cancer [48]. A potential role for β-catenin has been described in CLDN1-regulated anoikis resistance in gastric cancer cells, with β-catenin overexpression also reactivating Akt and Src signaling [48]. Interestingly, the contribution of CLDNs to anoikis resistance, and their potential therapeutic druggable potential are highlighted by the antitumor effect of the quinazoline-based doxazosin derivative DZ-50 [49]. Indeed, this agent interferes with tumor growth and metastasis via sensitizing cells to anoikis, by targeting key functional intercellular interactions, focal adhesions, and tight junctions in prostate cancer. In this context, treatment with DZ-50 resulted in decreased cell survival, migration and adhesion to extracellular matrix components, with one of the primary downregulated targets of DZ-50 being CLDN11 [49]. It is worthy to mention that a pro-apoptotic role of CLDNs and occludin has also been reported [42]. For example, the number of apoptotic cells was decreased upon intratumoral injection of CLDN3 siRNA into OVCAR-3 xenografts [38]. This differential functionality could be related to the experimental model or the pathophysiological state in which these proteins were studied. Indeed, similar pro- and anti-apoptotic roles were reported for other molecules, for instance nitric oxide, where its dual contrasting apoptotic functions were dependent on its concentration, flux and cell type [50].

Beside descriptive findings, the interrelation between tight junction dynamics and intracellular signaling pathways that control cell cycles and apoptosis has not been fully explored. This highlights the unmet need to study the functional links between TJ modulation and prominent pathways that regulate proliferation and apoptosis, to link extracellular signals to transcription factors in the nucleus. These mainly include the MAPK/Ras/Raf/ERK, PI3K/Akt, Janus kinase/signal transducer and activator of transcription (JAK/STAT), wingless-related integration site (Wnt), and TGF-β pathways [51]. However, this is complexified by the functional crosstalk between these transduction cascades that not only regulate cell death and proliferation, but also converge to modulate invasion, metastasis and cellular plasticity, as discussed in the succeeding section.

### 3.2. Members of the TJ Protein Family as Regulators of Cell Migration and Plasticity

Abnormal TJ protein expression is linked to changes in cell plasticity and differential induction or suppression of EMT, thus highlighting these proteins as major regulators of invasion and metastasis [52]. In this context, CLDN1 expression was linked to enhanced invasiveness and metastasis of multiple malignancies, such as gastric carcinoma [53] and colon cancer [23,54]. Moreover, CLDN1 was described to promote mesenchymal transformation in hepatocellular carcinoma (HCC) by upregulating Slug and Zeb1 [55], and to enhance the invasive ability of oral squamous cell carcinoma by promoting the cleavage of laminin-5 gamma2 chains via MMP-2 and membrane-type MMP-1 [56]. Indeed, it is well conceived that CLDNs enhance cell invasion via the activation of MMPs [57]. In particular, CLDN1 activation of MMP-2 is mediated through the stimulation of the protein kinase C (PKC) signaling pathway in a panel of melanoma cell lines [58]. CLDN6 was also shown to induce MMP-2 activation through CLDN1. The latter is described to interact with the extracellular proMMP-2 through its ECLs, resulting in its activation by MMP-14 in human adenocarcinoma gastric cancer cells [59]. CLDN2, on the other hand, was described to increase the mRNA level and enzymatic activity of MMP-9 through elevating Sp1 nuclear distribution in the human lung adenocarcinoma cell line A549 [60]. In turn, proteolytically active MMPs not only degrade the ECM, but also form new cell–matrix and cell–cell attachments and change the adhesive phenotype of tumor cells toward EMT [61]. Beside proteases, CLDN7 expression was described to regulate E-cadherin expression and invasion in esophageal squamous cell carcinoma cells [62]. In addition, CLDN7 is required to recruit EpCAM for TACE/presenilin2, resulting in the generation of the EpCAM-cleaved intracellular domain, EpIC. The latter is responsible of the induction of EMT-associated transcription factor expression [63]. This broadens the conceptual framework for the mechanisms by which TJ proteins modulate invasion.

On the other hand, although CLDN4 overexpression increased the migration of breast cancer cells [39], the invasiveness and metastatic potential of pancreatic cancer cells was shown to be decreased upon CLDN4 expression [64]. In addition, CLDN7 downregulation may promote invasion and metastasis of colorectal cancer [65], and was positively correlated with the depth of invasion, lymphatic vessel invasion and lymph node metastasis in esophageal squamous cell carcinoma [66]. This positive correlation was also described for venous invasion and liver metastasis in the context of colorectal cancer [67] and distant metastases in high-grade serous ovarian carcinoma patients [68]. MarvelD3 was transcriptionally downregulated during mesenchymal transition in pancreatic cancer cells [69], whereas its expression inhibits EMT, along with NF-κB pathway inactivation, a main regulator of EMT and cell metastasis [70]. In line with these findings, MarvelD3, a dynamic regulator of the MEKK1–c-Jun NH_2_-terminal kinase (JNK) pathway, was described to reduce pancreatic cancer cells’ tumor formation in vivo and Caco-2 cells’ proliferation and migration. Indeed, MarvelD3 recruits MEK kinase 1 (MEKK1), an MAPK kinase, leading to the downregulation of JNK phosphorylation. Subsequently, this inhibits JNK-mediated transcriptional mechanisms that regulate cell behavior, including migration [71]. This highlights the fact that the altered expression of TJ proteins in various cancer types and tissues might differentially modulate cell migration, invasion and metastasis, with a potential disparity in transduction pathway activation. Despite this, it is well reported that these TJ proteins act as a pivot for intracellular signaling pathways that modulate metastasis in several malignancies [52], as detailed in the following section.

***TGF-β-dependent pathway signaling***. Among the pathways established to induce EMT, canonical SMAD-dependent TGF-β signaling is described to be a major driver [72]. In fact, TGF-β-induced cell migration is linked to the induction of CLDN1 expression in ovarian cancer cells [73]. Additionally, the RNA-binding motif protein 38 (RBM38), a pivotal mediator of TGF-β-induced EMT, positively regulates ZO-1 transcription via direct binding to AU/U-rich elements in its mRNA 3′-UTR in breast cancer [74]. On the other hand, SMAD2 suppresses CLDN6 expression through DNMT1-mediated methylation of CLDN6 promoter, thus promoting cell migration and invasion in breast cancer [75]. Beside direct modulation, CLDNs can contribute to the release of active TGF-β, thus enhancing EMT. For example, CLDN1 activates the membrane-type 1 matrix metalloproteinase, as mentioned previously [56], which is responsible for the proteolytical release of TGF-β from the subendothelial matrix [76]. Similarly, TGF-β1, its receptor, and receptor-mediated signaling are partly activated by MMP-2, also induced by CLDN1 [77].

***Ras-Raf-MEK-ERK and PI3K/Akt signaling.*** TGF-β-induced EMT can also occur through non-SMAD pathways, for instance Ras-Raf-MEK-ERK and PI3K/Akt pathways [72]. The c-Abl-Ras-Raf-1-ERK1/2 signaling axis was shown to be activated upon CLDN1 expression, with the subsequent upregulation of Slug and Zeb1 and EMT induction in Chang cells [55]. In this context, PKCα regulated CLDN1 expression via Snail- and MAPK/ERK-dependent pathways during EMT in human pancreatic cancer, shedding the light on the potential therapeutic status of PKCα inhibitors [78]. In line with these findings, Snail, but not Zeb1 nor Twist1, was also highlighted for its CLDN6-mediated invasive abilities in gastric cancer. Indeed, CLDN6 increased YAP1 nuclear translocation, which enhanced the interaction between YAP1 and Snail1 to promote EMT [79]. Furthermore, Snail enhanced the migration of squamous cell carcinoma by inducing the expression of CLDN11 through the tyrosine-mediated phosphorylation of the latter, which activated Src and suppressed RhoA activity [80]. CLDN6 also enhanced endometrial carcinoma cell migration via the PI3K/AKT/mTOR signaling pathway [81]. Junctional adhesion molecule-A (JAM-A) led to EMT via the activation of the PI3K/Akt pathway in human nasopharyngeal carcinoma [82]. On the other hand, expression of CLDN1 inhibited the migration potential of human osteosarcoma cells through the inhibition of the Ras/Raf/MEK/ERK signaling pathway [83]. Furthermore, CLDN3 and CLDN4 impeded EMT in ovarian carcinoma through the activation of the PI3K/Akt pathway [84], in line with CLDN7-mediated inhibition of cell migration and invasion through the ERK/MAPK signaling pathway in human lung cancer cells [85]. In parallel, CLDN18 suppressed human lung adenocarcinoma cell motility by inhibiting the PI3K/PDK1/Akt signaling pathway [86]. In addition, it has been shown that occludin downregulation in the context of Ras-Raf-driven epithelial transformation might play an essential role in mediating the loss of the structure and function of epithelial TJs through the MEK-ERK signaling pathway [29]. This differential regulation of cell behavior highlights the context-dependent role of TJ proteins as inducers or inhibitors of EMT in carcinogenesis.

***Wnt/β-catenin/T-cell and lymphoid enhancer (TCF-LEF) signaling***. Dysregulation of Wnt/β-catenin signaling can trigger the induction of EMT, which could lead to metastasis. This is mainly mediated by β-catenin nuclear translocation and binding to TCF-LEF factors, which activates the transcription of target genes with a pro-invasive expression profile [87]. Smad4 is a central intracellular signal transduction component of the TGF-β family that was described to mediate invasion suppressive effects in colon cancer via the modulation of β-catenin/TCF-LEF activity, resulting in the repression of CLDN1 transcription [88]. This is further confirmed by the positive correlation of CLDN1 expression levels with β-catenin levels in gastric cancer [89]. Furthermore, CLDN3-modulated EMT is mediated through the regulation of the Wnt/β-catenin signaling pathway in lung squamous cell carcinoma [90], a role also shared by CLDN4 [91]. CLDN7 expression is significantly correlated with lymph node metastasis in salivary adenoid cystic carcinoma, where its expression regulates metastasis also through the modulation of Wnt/β-catenin signaling [92].

***STAT signaling.*** The activity of a multitude of master EMT transcription factors that function to stimulate rapid transitions between epithelial and mesenchymal phenotypes is regulated by STAT3 [93]. CLDN9 was shown to promote the invasive behavior of hepatocytes in vitro, by activating the Tyk2/STAT3 signaling pathway [94]. On the other hand, Tyk2/STAT1 signaling was described in CLDN12-induced EMT in lung squamous cell carcinoma [95].

Beside signaling pathways, the localization of tight junction proteins is also altered during EMT. Enhanced non-junctional CLDN1 expression, e.g., in the nucleus or cytoplasm, was detected in colon carcinomas and metastatic lesions, in contrast to cell membrane-restricted staining in normal colonic mucosa [23]. It is worth mentioning that besides single tumor cell metastasis, collective cell migration is also described as a fundamental process where migrating clusters maintain cell-to-cell junctions and exhibit a higher invasive capacity and therapeutic resistance compared to single cell migration [96]. In this context, the aberrant expression of CLDN1 was shown to support collective cell migration [97]. In addition, CLDN11 was described to prompt the formation of circulating tumor cell clusters through the activation of a Snail–CLDN11 axis in head and neck cancers [80]. In line with these findings, the overexpression of CLDN11 and occludin was described to enhance the collective migration of peritumoral cancer-associated fibroblasts via TGF-β secretion [98,99].

Whether CLDNs retain the junctions between cancer cells or form cell adhesion complexes to enhance the metastatic efficiency remains unclear. Further studies are needed to decipher the additional aspects regarding the mechanisms by which TJ proteins modulate the modes of EMT-mediated cancer migration. This can be further clarified by coupling the study of the altered signaling pathways to the localization of the dysregulated TJ proteins. Importantly, and in the light of the heterogenic roles of TJ components at different stages of the metastatic cascade, context-dependent and individual assessment of these proteins’ role could reveal novel therapeutic targets. As many of the migration and EMT studies cited above have been conducted in conventional cell culture systems, the adoption of experimental models that reflect better physiological or pathological characteristics raises a crucial unmet need.

### 3.3. Functional Role of Tight Junction Proteins in Cancer Cell Stemness

With their first identification in 1994, cancer stem cells (CSCs) emerged as a critical cancer subpopulation subset endowed with tumor-initiating properties, self-renewal abilities and multi-lineage differentiation, opening the door toward tumor heterogeneity and therapeutic resistance [100].

A growing body of evidence points toward the important role of tight junction proteins, in particular CLDNs, in cancer stem-like cell biology [101]. In this setting, claudin functions are regulated at various levels, and by distinct mechanisms. cDNA microarray analysis identified CLDN1 as one of the most significantly upregulated genes in ovarian cancer-initiating cells [102]. Moreover, CLDN1 overexpression was shown to induce dedifferentiation in primary colon adenocarcinoma [23]. CLDN2 promoted the self-renewal of colon cancer cells in vitro, as well as colorectal cancer self-renewal in vivo, along with increasing the population of ALDH^High^ stem-like cells and favoring phenotypic transitions from ALDH^Low^ toward ALDH^High^ subpopulations [99]. Furthermore, CLDN3 was uncovered as a positive regulator of cancer stemness in non-squamous non-small cell lung carcinoma, where stemness suppression and chemoresistance reversal were observed upon CLDN3 transcriptional activity downregulation [103]. In contrast, CLDN1 depletion increased the invasive and CSC-like properties of hepatocellular carcinoma cell lines [104]. In line with these findings, CLDN7 deficiency was shown to confer stemness properties and to promote tumor-initiating cell features in colorectal cancer stem cells [22].

CSC regulation is complex and multiple intracellular signaling pathways and extracellular factors have been shown to be implicated. Of those, the Hedgehog (Hh) and Notch pathways are highlighted as key signals in this specific cell phenotype [105].

***Hedgehog pathway***. As an evolutionarily conserved pathway that is essential for cell fate determination, the aberrant activation of the Hh pathway serves as a crucial asset for CSC function and maintenance during tumorigenesis [106]. In this context, the expression of CLDN3, CLDN5, occludin, and JAM-A was increased in response to the activation of Hedgehog signaling. In contrast, treatment with cyclopamine, an Hh pathway inhibitor, decreased the expression of these proteins [107]. Cyclopamine also downregulated CLDN4 and occludin expression in colon cancer stem cells [108]. Importantly, CLDN1 is pinpointed as a direct transcriptional target of Hh pathway activation in breast cancer, as evidenced by the correlation between membranous CLDN1 expression and Hh paracrine pathway activation [109].

***Notch pathway***. Beside the Hh pathway, CLDN1 was identified as one of the dynamic regulators of Notch signaling [25]. CLDN1 overexpression increased Notch and Wnt signaling at the transcriptomic level, as evidenced by an increase in Hes1 and a decrease in Math1 expression in a mouse colon cancer model [110]. Indeed, CLDN1 upregulation activates Notch signaling in parallel to the induction of MMP-9 expression and p-ERK signaling, thus interfering with cellular differentiation, and enhancing susceptibility to mucosal inflammation and hyperplasia [25]. Beside the mentioned mechanisms, upregulated CLDN1 expression was shown to promote Notch signaling through its noncanonical role in regulating Notch/PI3K/Wnt/β-cateninSer552 signaling, which underlies the induction of colitis-associated cancer [26]. Interestingly, this CLDN1/Notch axis could be therapeutically targeted by CLDN1-specific monoclonal antibodies, with the latter resulting in the inhibition of Notch cleavage in HCC cell-based and CDX animal models [111]. Moreover, the Notch signaling pathway was identified as one of the pathways that is regulated by CLDN5 in the context of lung cancer brain metastasis [112]. In addition, upregulated Notch expression in holoclones was associated with CLDN7 expression in colon adenocarcinoma [113].

***Miscellaneous pathways***. Other pathways are also implicated in CSC biology [114]. For example, the CLDN2-dependent regulation of stem-like cell self-renewal is mediated through the activation of YAP and downstream repression of miR-222-3p [99]. Moreover, indirect protein interaction was reported between CLDN7 and SOX9, a vital player in CSC self-renewal and a master regulator of several stem cell markers [22]. The human growth hormone (hGH)-STAT3-CLDN1 axis was described to be responsible for invasive and CSC-like properties in HCC [104]. Furthermore, CLDN1 regulation during ovarian cancer-initiating cell proliferation and invasion was shown to be mediated by miR-155, where the endogenous mature form of the latter may inhibit cancer-initiating cell growth via reducing CLDN1 expression by targeting its mRNA on the 3’-UTR [115].

Beside the potential role of TJ proteins in the regulation of CSC cell biology, a subsequent correlation with poor prognosis, therapeutic resistance and relapse could be speculated, all of which being key characteristics of CSCs [100]. Indeed, multiple tight junction proteins were highlighted as potential biomarkers in the prognostic outcome of cancer patients. For example, elevated JAM-A expression is significantly correlated with poor prognosis in breast cancer patients [116]. Partitioning defective protein 3 (Par3) and ZO-1 clustering on the cell membrane are indicators of poor prognosis in lung squamous cell carcinoma [117]. Moreover, CLDN1 is correlated with a poor prognosis of oral squamous cell carcinoma [118] and lung adenocarcinoma [119]. The poor prognosis of gastric and breast cancer patients is associated with CLDN4 overexpression, in line with the poor prognostic value of CLDN7 in gastric cancer [120,121,122].

On the other hand, the development of therapeutic resistance is a major challenge facing cancer therapy, in which CSCs play an important role [100]. Various mechanisms are employed by TJ proteins to mediate chemoresistance, including their effects on apoptosis and autophagy, as well as on drug transporters. In this setting, cisplatin resistance in non-small cell lung cancer was shown to be promoted by CLDN1-induced activation of autophagy via the activation of ULK1 phosphorylation [123]. Doxorubicin resistance was also reported in lung adenocarcinoma cells, where CLDN1 is speculated to inhibit the penetration of anticancer drugs into the target area [124]. CLDN4 knockdown increased cellular accumulation and sensitivity to cisplatin, pointing towards the potential involvement of CLDN4 in platinum resistance in ovarian cancer [125], in line with the enhanced sensitivity toward carboplatin and paclitaxel upon CLDN1 knockdown in ovarian cancer cells [126]. This could be partly explained by the interactions of some CLDNs with the microtubule network, notably tubulin, resulting in the re-shaping of its structure and polymerization toward a reduced apoptotic response to the microtubule-targeting paclitaxel [127]. Alternatively, relapse-free survival (RFS) was significantly shorter in high versus low CLDN2 or CLDN5 expression in breast cancer [128,129], with high CLDN4 expression also being associated with worse RFS [121]. Furthermore, the substantial association between high CLDN2 expression in cancer-associated fibroblasts and shorter survival in 5-fluorouracil- and oxaliplatin-treated metastatic colorectal cancer patients has been reported [130]. In addition, cytoplasmic CLDN3 and CLDN7 expression was associated with poor RFS in triple-negative breast cancer [131]. In the context of HCC, CLDN10 expression was underlined as a molecular marker of disease recurrence after curative hepatectomy [132]. Nevertheless, various studies have reported a negative correlation between tight junction protein expression and poor prognosis, therapeutic resistance, or disease relapse [101]. Furthermore, contradictory findings were also reported for the same CLDN molecule. For example, in renal cell carcinoma, CLDN1 expression was correlated with shortened disease-specific patient survival; however, an opposite finding was described for papillary renal cell carcinoma [133]. This emphasizes the heterogenous nature of tumors and the potential differential expression pattern of tight junction proteins during distinct stages of tumor development, as well as cellular differentiation. This requires a careful assessment of these molecules, as the interplay between cancer stem cell enrichment and TJ proteins can reveal potential clinical perspectives of the latter as clinicopathologic parameters or prognostic factors.

### 3.4. TJ Proteins and Epigenetic Regulation in Cancer

In a recent update published in 2022, Hanahan proposed “nonmutational epigenetic reprogramming” as a new distinctive enabling characteristic that expedites the acquisition of cancer hallmark capabilities during tumor development and progression [134], highlighting the importance of these modifications in oncogenic transformation. In this setting, various epigenetic mechanisms are reported to regulate tight junction protein expression in favor of malignant transformation.

Although playing an essential role in biologic processes, aberrant promoter methylation is extensively associated with carcinogenesis [135]. The CpG island hypermethylation of occludin promoter enhances the tumorigenic, invasive, and metastatic properties of cancer cells [16]. JAM-3 is frequently downregulated in colorectal cancer through DNA methylation [136], with the latter also silencing CLDN1 [137]. CLDN3 promoter hypermethylation is described in HCC and advanced gastric adenocarcinoma [138,139], as well as the hypermethylation of CLDN11 promoter in melanomagenesis [140]. On the other hand, CLDN4 upregulation during early gastric tumorigenesis is strongly associated with DNA hypomethylation, along with decreased repressive H3K27me3 and H4K20me3 histone marks, and an increased active H3K4me3 and H4Ac histone marks [141]. CLDN5 is also identified as an aberrant methylation target in pancreatic carcinoma [142], as well as CLDN6 in breast and esophageal squamous cell carcinoma [143,144], and CLDN7 in breast ductal [145] and colorectal carcinoma [146]. Interestingly, differential CLDN4 expression reported as CLDN4 overexpression in differentiated carcinomas compared to the downregulation in invasive/high-grade bladder tumors was associated with a low versus high level of CLDN4 methylation, respectively [147]. This is also noted with CLDN1, with an increased promoter methylation-expression pattern in recurrent ovarian cancer, compared to the primary cancer [126]. In addition, CLDN1 promoter CpG island methylation was relatively frequent in estrogen receptor-positive breast cancer, but not estrogen receptor-negative samples [148]. This could point towards a complex and differential epigenetic-mediated expression pattern of TJ proteins in different tumor stages and grades, highlighting their potential as promising prognostic markers. In fact, CLDN1 has been described as a strong prognostic indicator of disease recurrence and poor patient survival in stage II colon cancer [149]. Furthermore, the combined low expression of CLDN3, -4, -7 and -8 in metaplastic and basal-like breast cancer is proposed to be a strong predictor of disease recurrence [150].

Epigenetic modulation of tight junction proteins is best demonstrated through treatment with epigenetic modulators/inhibitors. Indeed, AZA, a DNA methylation inhibitor, was found to downregulate CLDN2 expression [34]. Interestingly, in this context, epigenetic modulation could intersect with intracellular signaling pathways, in addition to regulating gene transcription. For instance, it was reported that CLDN2 is downregulated through the inhibition of Akt and NF-κB phosphorylation by AZA [34]. Indeed, interactions between the epigenetic machinery and various signaling pathways, including MAPK, Notch, Wnt, JAK/STAT, NF-κB and JNK pathways, have been described [151]. It could be of interest to link epigenetic modifications to signaling pathways in order to broaden our understanding of the complex, yet intersecting, mechanisms by which a cell induces transcriptional changes in response to external and internal signals during carcinogenesis. In this context, special attention should be paid to the dynamic nature of the tumor microenvironment, as it is correlated with the epigenetic re-programming and the activation of tumor-promoting signaling cascades by harboring various hormones and growth factors. For example, estrogen stimulation upregulates CLDN1 expression in cervical adenocarcinoma via G protein-coupled receptor 30 through ERK and/or Akt signaling [152].

Taken together, signaling mediated by members of the TJ protein family plays a key role in the pathogenesis of solid tumors and is associated with tumor initiation, progression, and metastasis. These functional effects correlate with differential subcellular delocalization and expression patterns. However, the complexity and plasticity associated with the functions of TJ proteins in the mediation of tumorigenesis go beyond signaling pathway perturbations. This includes the loss of membrane polarity via tight junction abnormalities, increased paracellular permeability and junctional remodeling. The resulting dysregulation of this machinery can have deleterious effects not only on cellular homeostasis, but also on the interactions with the extracellular matrix. Coupling the perturbations in cell-to-cell adhesion to adhesion-independent signal transduction perturbations will broaden our understanding of how TJ molecules contribute to cancer pathogenesis.

## 4. Members of the Tight Junction Protein Family as Targets for Cancer Treatment

Considering the critical role of tight junctions in carcinogenesis, these proteins are currently perceived as potential therapeutic targets, with multiple approaches being employed, ranging from monoclonal antibodies (mAbs) and targeting molecules, to therapeutic gene delivery [13].

Antibodies that target the TJ components have been recently described, with their pharmaceutical activities being explored in experimental and clinical settings. Importantly, this therapeutic targeting is not limited to junctional proteins, but has also been applied to exposed TJ proteins located outside the TJs. Overexpression of non-junctionally exposed CLDN1 has been described at the basolateral membrane of human hepatocytes during advanced liver fibrosis and hepatocellular carcinoma, where CLDN1 mediates key regulatory cell functions, such as differentiation, proliferation, and migration, by recruiting signaling proteins in response to extracellular stimuli [153]. Non-junctionally exposed CLDN1 serves as a cell entry factor of HCV—a major cause of liver cancer worldwide [154]. Interestingly, treatment with humanized monoclonal antibodies (mAbs) that selectively target the extracellular loop 1 of non-junctionally exposed CLDN1 suppressed liver cancer growth and EMT in patient-derived ex-vivo models and reprogrammed the tumor microenvironment in patient-derived HCC spheroids, an effect also confirmed in in vivo proof of concept CDX and PDX models [155]. Inhibition of cancer growth and invasion was mainly mediated through interference with oncogenic signaling pathways, notably the Notch cascade [111]. In addition, these highly specific mAbs demonstrated significant and robust antitumoral effects in vivo across cell line-derived and patient-derived xenograft models for intra- and extrahepatic cholangiocarcinoma [156]. Safety studies on non-human primates showed no detectable adverse events even at high steady-state concentrations, hence providing a preclinical proof-of-concept for CLDN1-specific mAbs for liver cancer prevention and treatment [157]. Collectively, these data provide an opportunity for the clinical development of CLDN1-specific antibodies for liver cancer. Given the high expression of CLDN1 in other solid tumors [110,158,159], CLDN1-targeting approaches may be used to treat a broad range of solid tumors. On the other hand, treatment with the human–rat chimeric IgG1 1A2 CLDN2-targeting antibody attenuated fibrosarcoma tumor growth without remarkable side effects [160]. The anti-CLDN4 extracellular domain antibody 4D3 synergistically enhanced the antitumoral effects of 5-fluorouracil or the anti-EGFR antibody C225 (cetucimab) in colorectal cancer [161]. KM3900, a monoclonal antibody that recognizes the extracellular loop 2 of CLDN4, induced antibody-dependent cellular cytotoxicity and complement-dependent cytotoxicity in vitro, while inhibiting pancreatic and ovarian tumor growth in SCID mice in vivo [162]. Similar anti-tumor activity was also noted upon treatment with KM3907, a dual-targeting monoclonal antibody against the extracellular loop 1 of CLDN3 and CLDN4 [163]. The anti-CLDN6 antibody IMAB027, also known as ASP1650, has been studied in women with recurrent advanced ovarian cancer [164], and men with incurable platinum refractory germ cell tumors [165]. The CLDN18.2-targeting antibodies zolbetuximab (IMAB362; claudixmab) and NBL-015 were also studied in gastro-esophageal cancer [166] and in patients with advanced solid tumors [167], respectively. Notably, NBL-015 was granted the status of orphan-drug designation (ODD) by the U.S. Food and Drug Administration (FDA) for the treatment of pancreatic and gastric cancers, including cancer of gastroesophageal junctions [167]. The ODD status has also been granted to I-Mab’s TJ-CD4B, a first clinical-stage bispecific antibody that binds to CLDN18.2 and the co-stimulatory molecule 4-1BB on T cells to exert a tumor-killing effect in the setting of gastric cancer [168]. Of note, the anti-JAM-C mAb H225 abolished mantle cell lymphoma cell engraftment in a xenograft model [169]. An injection of anti-JAM-A mAb 6F4 significantly inhibited the growth of epidermoid carcinoma xenograft models of MCF-7 and A431 cells [170]. Data that summarize the current results and status of anti-CLDN antibodies in clinical trials are reviewed in Table 1.

Moreover, engineered Clostridium perfringens enterotoxin (CPE)-related molecules, including toxin-conjugated CPE fragments, have demonstrated antitumor effects. For instance, an injection of a CLDN4-targeting molecule, consisting of the fusion of the C-terminal fragment of CPE (C-CPE) and the protein synthesis inhibitory factor (PSIF) derived from Pseudomonas aeruginosa exotoxins, reduced tumor growth in vivo [174]. Another fusion molecule that targets CLDN4 in ovarian cancer cells was also described, with CPE being fused to TNF at its NH(2)-terminal end [175]. Targeted gene therapy of CLDN3 and/or four overexpressing colon cancer cell lines was also described through the use of an optimized CPE-expressing vector that functions as targeted suicide gene therapy, with the latter resulting in rapid and effective tumor cell killing in vitro and in vivo [176]. Nevertheless, CPE immunogenicity and potential toxicity might limit its clinical applications [177]. This could be overcome with local administration, or by adopting alternative technologies, such as the development of monoclonal antibodies that target these TJ proteins, as mentioned above. Other treatment modalities for cancer immunotherapy include the incorporation of a panel of engineered CLDN6 variants into the membrane of retrovirus-derived virus-like particles (VLPs), eliciting complement-dependent cytotoxicity in solid tumors [178]. The latter was also noted upon the administration of a combination of the measles virus and the CLDN6 tumor vaccine [179].

Of note, the anti-JAM-C antibody antitumoral effect was mediated through the inhibition of ERK1/2 phosphorylation [169]. Superior attention to the signaling pathways implicated needs to be granted to thoroughly understand the molecular mechanism of action that underlies the effectiveness of those therapeutic tools. In particular, this could allow the potential repositioning of some of these therapeutic antibodies in the setting of uncurable or resistant malignancies.

## 5. Conclusions and Perspectives

A large body of research in the last decade has shown that TJ proteins are not only statically expressed in tight junctions to contribute to barrier function, but are dynamically involved in a wide array of cellular processes that regulate proliferation, migration, plasticity, and differentiation, all of which are central to cancer initiation and progression. The differential expression of TJ proteins in cancer, combined with the gain and loss of functions, has unveiled their important functional role in carcinogenesis in a tissue- and context-dependent matter, as well as during diverse stages of cancer progression, including invasion, metastasis, or relapse. Pre-clinical and clinical studies that target several members of the TJ protein family have reported the targetable properties of these molecules, as well as their safety and efficacy. Furthermore, recent clinical studies using monoclonal antibodies have demonstrated that TJ proteins are a valuable target to improve the outcome of solid tumors.

## Figures and Tables

**Table 1:** Anti-CLDN antibodies and their therapeutic effects in clinical trials.

Antibody	Target	Clinical Trial Results	References
IMAB027 (ASP1650)	Extracellular loop 2 of CLDN6	Safe and well tolerated in women with recurrent advanced ovarian cancer; well tolerated but lacked efficacy in male patients with relapsed refractory germ cell tumors	[164,165,171]
BNT211	CLDN6	Multi-center open-label trial demonstrated a favorable safety profile at the doses tested with encouraging signs of efficacy	[172]
IMAB362 (claudiximab; zolbetuximab)	CLDN18.2 (mouse–human chimeric IgG1 antibody)	- Multiple open-label phase II clinical trials demonstrated inhibition of tumor growth by IMAB362 monotherapy or combination therapy in gastro-esophageal cancer. Multiple other phase II and III trials are ongoing - Phase III clinical trial demonstrated significant progression-free survival in locally advanced unresectable or metastatic gastric or gastroesophageal junction adenocarcinoma	[166,173]
NBL-015	CLDN18.2 (humanized antibody)	Open-label, multi-center, dose-escalation phase I clinical study in patients with advanced solid tumors started in 2021	[167]

## Data Availability

Not applicable.
